# Metal–nucleobase inter­action: bis[4-amino­pyrimidin-2(1*H*)-one-κ*N* ^3^]dibromidozinc(II)

**DOI:** 10.1107/S1600536810049305

**Published:** 2010-11-30

**Authors:** Ammasai Karthikeyan, Samuel Ebenezer, Packianathan Thomas Muthiah

**Affiliations:** a School of Chemistry, Bharathidasan University, Tiruchirappalli 620 024, Tamilnadu, India

## Abstract

In the title complex, [ZnBr_2_(C_4_H_5_N_3_O)_2_], the central metal ion is coordinated to two bromide ions and endocyclic N atoms of the two cytosine mol­ecules leading to a distorted tetra­hedral geometry. The structure is isotypic with [CdBr_2_(C_4_H_5_N_3_O)_2_] [Muthiah *et al.* (2001). *Acta Cryst.* E**57**, m558–m560]. There are two inter­ligand N—H⋯Br hydrogen bonds, generating two hydrogen-bonded rings stabilizing the coordination sphere. The complex aggregates, forming supra­molecular chains, sheets and staircases through N—H⋯O and N—H⋯Br hydrogen bonding and π–π stacking inter­actions [centroid–centroid distance = 3.616 (2) Å].

## Related literature

For metal ion–nucleic acid inter­actions, see: Muller (2010 ▶). For different modes of binding between metal ions and cytosine, see: Lippert (2000 ▶). For an isotypic complex, see: Muthiah *et al.* (2001 ▶).
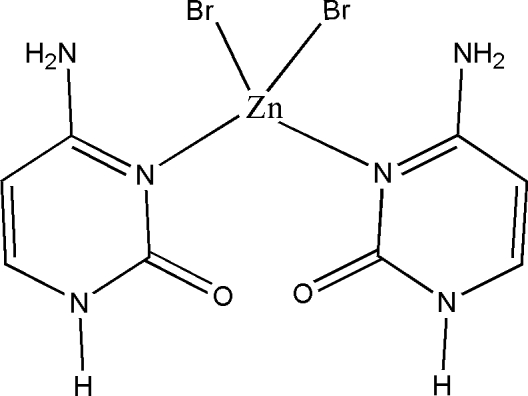


## Experimental

### 

#### Crystal data

[ZnBr_2_(C_4_H_5_N_3_O)_2_]
*M* *_r_* = 447.41
Triclinic, 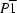

*a* = 7.1337 (2) Å
*b* = 7.8375 (2) Å
*c* = 12.4275 (3) Å
α = 86.746 (2)°
β = 75.199 (2)°
γ = 87.448 (2)°
*V* = 670.36 (3) Å^3^
*Z* = 2
Mo *K*α radiation
μ = 7.80 mm^−1^
*T* = 293 K
0.3 × 0.2 × 0.2 mm

#### Data collection

Bruker SMART APEXII CCD area-detector diffractometer
Absorption correction: multi-scan (*SADABS*; Bruker, 2008 ▶) *T*_min_ = 0.203, *T*_max_ = 0.305
13254 measured reflections
2973 independent reflections
2204 reflections with *I* > 2σ(*I*)
*R*_int_ = 0.043

#### Refinement

*R*[*F* ^2^ > 2σ(*F* ^2^)] = 0.036
*wR*(*F* ^2^) = 0.083
*S* = 1.02
2973 reflections
172 parameters
H-atom parameters constrained
Δρ_max_ = 0.69 e Å^−3^
Δρ_min_ = −0.44 e Å^−3^

### 

Data collection: *APEX2* (Bruker, 2008 ▶); cell refinement: *SAINT* (Bruker, 2008 ▶); data reduction: *SAINT*; program(s) used to solve structure: *SIR92* (Altomare *et al.*, 1993 ▶); program(s) used to refine structure: *SHELXL97* (Sheldrick, 2008 ▶); molecular graphics: *PLATON* (Spek, 2009 ▶); software used to prepare material for publication: *PLATON*.

## Supplementary Material

Crystal structure: contains datablocks global, I. DOI: 10.1107/S1600536810049305/hg2756sup1.cif

Structure factors: contains datablocks I. DOI: 10.1107/S1600536810049305/hg2756Isup2.hkl

Additional supplementary materials:  crystallographic information; 3D view; checkCIF report

## Figures and Tables

**Table 1:** Hydrogen-bond geometry (Å, °)

*D*—H⋯*A*	*D*—H	H⋯*A*	*D*⋯*A*	*D*—H⋯*A*
N1*A*—H1*A*⋯O2*A*^i^	0.86	1.94	2.766 (5)	161
N1*B*—H1*B*⋯Br1^ii^	0.86	2.70	3.483 (3)	151
N4*A*—H2*A*⋯Br1	0.86	2.74	3.577 (4)	165
N4*B*—H2*B*⋯Br2	0.86	2.65	3.454 (3)	155
N4*A*—H3*A*⋯Br2^iii^	0.86	2.91	3.339 (4)	112
N4*B*—H3*B*⋯O2*B*^iv^	0.86	2.19	3.003 (5)	157
C5*A*—H5*A*⋯Br2^v^	0.93	2.87	3.726 (4)	153
C6*A*—H6*A*⋯O2*B*^vi^	0.93	2.42	3.292 (6)	156

Symmetry codes: (i) 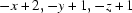; (ii) 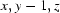; (iii) 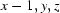; (iv) 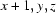; (v) 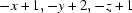; (vi) 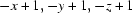.

**Fig. 1.**
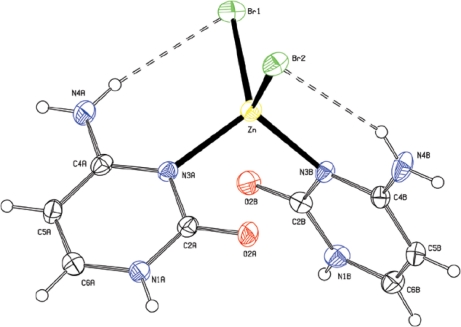
The asymmetric unit of (I), showing 50% probability displacement ellipsoids. Dashed lines indicate hydrogen bonds.

**Fig. 2.**
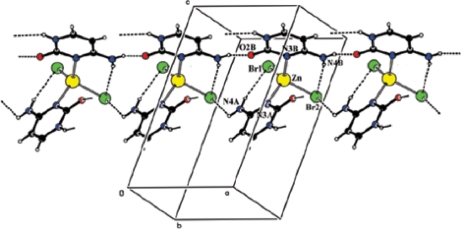
View of an infinite chain linked by N4B—H4B···O2B and N4A-H4A2···Br2 hydrogen bonds.

**Fig. 3.**
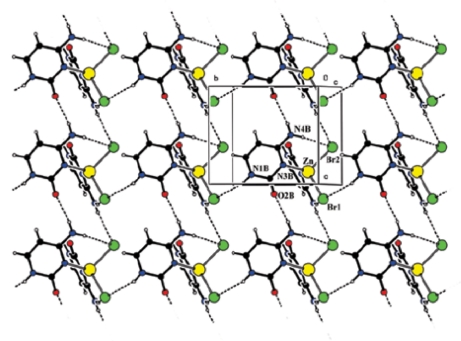
View of a supramolecular sheet along the (001) plane.

**Fig. 4.**
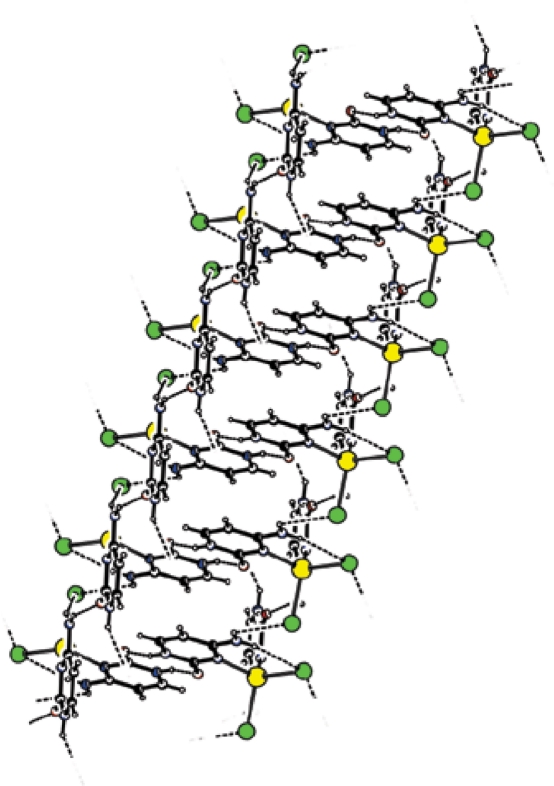
Molecular staircase formed by pairing of two infinite chains through hydrogen bonding and stacking interactions.

## supplementary crystallographic information

### Comment

The studies of metal ion–nucleic acid interactions are of continued interest in bioinorganic chemistry (Muller, 2010). There are several modes of binding between a cytosine and metal ion. The cytosine coordinates in a monodentate fashion either through N3, N4, O2 or C5 sites. Similarly it acts as a bidentate ligand by chelating, semi-chelating or bridging *via* N3, O2 and N3, N4 sites (Lippert, 2000). However the most preferable mode of binding is *via* N3 as observed in majority of the cases. In the present study we have prepared a metal complex of zinc-cytosine as a model for Zn (II) ion interactions with guanine-cytosine rich regions of nucleic acids (DNA and RNA). The crystal structure is found to be isomorphous with the earlier reported structure of dibromobis(cytosine)cadmium(II) (Muthiah *et al.*, 2001).

The title complex is coordinated by two bromide ions in addition to two cytosine molecules. The *ORTEP* view is shown in Figure 1. The two crystallographically independent cytosine molecules coordinate through N3 position forming a tetrahedral geometry around the central Zn (II) ion with slight distortion. This distortion is not only because of the dissimilar ligands coordinated to the central metal ion but is due to the additional attraction between the zinc ion and the oxygen of the cytosine molecule. This can be confirmed by looking into the contact distances between Zn···O in both the molecules (A and B) which are 2.804 (3) Å and 2.858 (3) Å respectively. It is further substantiated by the exocyclic bond angles at N3 (Zn—N3—C4 and Zn—N3—C2) of cytosine which is 132.0 (3)° and 109.0 (3)° for molecule A and 128.1 (3)° and 109.3 (2)° for molecule B. The stability of the coordinated metal complex is also enhanced by the two inter-ligand hydrogen bonds (N—H···Br hydrogen bond). These are formed between the amino group of the coordinated cytosine and the coordinated bromide ion which are lying in proximity. The interligand hydrogen bonds generate two hydrogen-bonded rings (Figure 1). These are very characteristic of metal-nucleobase interactions (Lippert, 2000).

The hydrogen bonding geometries of the title complex are given in Table 1. The two cytosines that have coordinated to the metal ion, although look similar, form different inter-molecular hydrogen bonds. The amino nitrogen of molecule B connects with the oxygen of the neighboring molecule *via* N4B—H4B···O2B extending into an infinite chain. This chain is supported by an additional weak hydrogen bond (N4A-H4A2···Br2) between the A molecules of neighboring cytosine (Figure 2). The infinite chain can further aggregate itself in two different ways. A supramolecular sheet is formed when the adjacent chains are linked by molecule B *via* N1B—H1B···Br1 hydrogen bonds (Figure 3). Similarly a staircase is formed when the inversely related chains pair up *via* N1A—H1A···O2A hydrogen bonds involving molecule A (Figure 4). These molecules form the steps of the staircase and stack one over the other through π-π stacking with a cg-cg distance of 3.616 (2) and a slip angle of 24.32°. Besides this, weak C—H···O and C—H···Br interactions are additionally present which stabilize the entire crystal structure.

### Experimental

Solution of zinc bromide anhydrous (0.056 g, 0.25 mmol) in 10 ml of hot propanol and cytosine (0.055 g, 0.50 mmol) in 10 ml of hot water were mixed mixed and dissolved in an 1:2 molar ratio. The resultant solution was heated over a water bath for an hour and on slow cooling the solution gave transparent colourless prismatic crystals.

### Refinement

All hydrogen atoms were positioned geometrically and were refined using a riding model. The N—H and C—H bond lengths are 0.86 and 0.93 Å respectively [*U*_iso_(H)=1.2 *U*_eq_ (parent atom)].

### Crystal data

**Table:**

[ZnBr_2_(C_4_H_5_N_3_O)_2_]	*Z* = 2
*M**_r_* = 447.41	*F*(000) = 432
Triclinic, *P*1	*D*_x_ = 2.217 Mg m^−^^3^
Hall symbol: -P 1	Mo *K*α radiation, λ = 0.71073 Å
*a* = 7.1337 (2) Å	Cell parameters from 2973 reflections
*b* = 7.8375 (2) Å	θ = 1.7–27.2°
*c* = 12.4275 (3) Å	µ = 7.80 mm^−^^1^
α = 86.746 (2)°	*T* = 293 K
β = 75.199 (2)°	Prism, colourless
γ = 87.448 (2)°	0.3 × 0.2 × 0.2 mm
*V* = 670.36 (3) Å^3^	

### Data collection

**Table:**

Bruker SMART APEXII CCD area-detector diffractometer	2973 independent reflections
Radiation source: fine-focus sealed tube	2204 reflections with *I* > 2σ(*I*)
graphite	*R*_int_ = 0.043
φ and ω scans	θ_max_ = 27.2°, θ_min_ = 1.7°
Absorption correction: multi-scan (*SADABS*; Bruker, 2008)	*h* = −9→9
*T*_min_ = 0.203, *T*_max_ = 0.305	*k* = −10→10
13254 measured reflections	*l* = −15→15

### Refinement

**Table:**

Refinement on *F*^2^	Primary atom site location: structure-invariant direct methods
Least-squares matrix: full	Secondary atom site location: difference Fourier map
*R*[*F*^2^ > 2σ(*F*^2^)] = 0.036	Hydrogen site location: inferred from neighbouring sites
*wR*(*F*^2^) = 0.083	H-atom parameters constrained
*S* = 1.02	*w* = 1/[σ^2^(*F*_o_^2^) + (0.0441*P*)^2^] where *P* = (*F*_o_^2^ + 2*F*_c_^2^)/3
2973 reflections	(Δ/σ)_max_ = 0.001
172 parameters	Δρ_max_ = 0.69 e Å^−^^3^
0 restraints	Δρ_min_ = −0.44 e Å^−^^3^

### Special details

**Table:**

Geometry. Bond distances, angles *etc*. have been calculated using the rounded fractional coordinates. All su's are estimated from the variances of the (full) variance-covariance matrix. The cell e.s.d.'s are taken into account in the estimation of distances, angles and torsion angles
Refinement. Refinement on *F*^2^ for ALL reflections except those flagged by the user for potential systematic errors. Weighted *R*-factors *wR* and all goodnesses of fit *S* are based on *F*^2^, conventional *R*-factors *R* are based on *F*, with *F* set to zero for negative *F*^2^. The observed criterion of *F*^2^ > σ(*F*^2^) is used only for calculating -*R*-factor-obs *etc*. and is not relevant to the choice of reflections for refinement. *R*-factors based on *F*^2^ are statistically about twice as large as those based on *F*, and *R*-factors based on ALL data will be even larger.

### Fractional atomic coordinates and isotropic or equivalent isotropic displacement parameters (Å^2^)

**Table:**

	*x*	*y*	*z*	*U*_iso_*/*U*_eq_	
Br1	0.34971 (6)	1.00965 (5)	0.84763 (4)	0.0378 (1)	
Br2	0.88031 (6)	1.05932 (5)	0.68592 (4)	0.0374 (1)	
Zn	0.63076 (7)	0.84924 (6)	0.74430 (4)	0.0303 (2)	
O2A	0.9020 (4)	0.6403 (4)	0.6017 (3)	0.0454 (11)	
O2B	0.4019 (4)	0.5579 (3)	0.8291 (3)	0.0372 (10)	
N1A	0.7973 (5)	0.6009 (4)	0.4478 (3)	0.0326 (11)	
N1B	0.5996 (5)	0.3618 (4)	0.8891 (3)	0.0364 (11)	
N3A	0.6105 (4)	0.7588 (4)	0.5954 (3)	0.0275 (10)	
N3B	0.6990 (4)	0.6425 (4)	0.8371 (3)	0.0264 (10)	
N4A	0.3118 (5)	0.8664 (4)	0.5860 (3)	0.0426 (12)	
N4B	1.0054 (5)	0.7147 (4)	0.8420 (3)	0.0439 (14)	
C2A	0.7755 (6)	0.6643 (5)	0.5505 (4)	0.0304 (12)	
C2B	0.5592 (6)	0.5230 (5)	0.8506 (3)	0.0296 (12)	
C4A	0.4734 (6)	0.7811 (5)	0.5385 (4)	0.0307 (14)	
C4B	0.8725 (6)	0.5973 (5)	0.8562 (3)	0.0283 (12)	
C5A	0.4985 (6)	0.7156 (5)	0.4323 (4)	0.0367 (16)	
C5B	0.9146 (6)	0.4288 (5)	0.8921 (4)	0.0352 (12)	
C6A	0.6632 (6)	0.6267 (5)	0.3895 (4)	0.0371 (16)	
C6B	0.7763 (7)	0.3150 (5)	0.9073 (4)	0.0396 (16)	
H1A	0.90110	0.54210	0.41930	0.0390*	
H1B	0.51060	0.28730	0.90240	0.0430*	
H2A	0.29720	0.90540	0.65110	0.0510*	
H2B	0.98030	0.81770	0.82100	0.0530*	
H3A	0.22130	0.88290	0.55190	0.0510*	
H3B	1.11690	0.68790	0.85380	0.0530*	
H5A	0.40390	0.73350	0.39310	0.0440*	
H5B	1.03500	0.39860	0.90470	0.0420*	
H6A	0.68450	0.58280	0.31920	0.0450*	
H6B	0.79990	0.20290	0.93040	0.0480*	

### Atomic displacement parameters (Å^2^)

**Table:**

	*U*^11^	*U*^22^	*U*^33^	*U*^12^	*U*^13^	*U*^23^
Br1	0.0296 (2)	0.0316 (2)	0.0490 (3)	−0.0027 (2)	−0.0031 (2)	−0.0055 (2)
Br2	0.0269 (2)	0.0323 (2)	0.0511 (3)	−0.0019 (2)	−0.0075 (2)	0.0042 (2)
Zn	0.0284 (3)	0.0287 (2)	0.0348 (3)	0.0000 (2)	−0.0101 (2)	−0.0002 (2)
O2A	0.0353 (18)	0.060 (2)	0.047 (2)	0.0205 (15)	−0.0223 (16)	−0.0176 (16)
O2B	0.0288 (17)	0.0358 (16)	0.0495 (19)	−0.0045 (13)	−0.0135 (14)	−0.0045 (14)
N1A	0.0248 (19)	0.0391 (19)	0.035 (2)	0.0063 (15)	−0.0093 (16)	−0.0096 (16)
N1B	0.039 (2)	0.0251 (17)	0.045 (2)	−0.0093 (16)	−0.0100 (18)	0.0027 (16)
N3A	0.0233 (18)	0.0268 (16)	0.0331 (19)	0.0014 (14)	−0.0089 (15)	−0.0022 (15)
N3B	0.0229 (18)	0.0254 (16)	0.0319 (19)	−0.0029 (14)	−0.0092 (15)	0.0026 (14)
N4A	0.032 (2)	0.050 (2)	0.050 (2)	0.0118 (18)	−0.0187 (19)	−0.0110 (19)
N4B	0.033 (2)	0.044 (2)	0.061 (3)	−0.0100 (17)	−0.0259 (19)	0.0166 (19)
C2A	0.028 (2)	0.030 (2)	0.034 (2)	0.0022 (18)	−0.0098 (19)	−0.0019 (18)
C2B	0.028 (2)	0.030 (2)	0.029 (2)	−0.0046 (18)	−0.0031 (18)	−0.0025 (18)
C4A	0.029 (2)	0.0242 (19)	0.039 (3)	−0.0022 (17)	−0.0098 (19)	0.0035 (18)
C4B	0.030 (2)	0.032 (2)	0.023 (2)	−0.0017 (18)	−0.0074 (17)	0.0017 (17)
C5A	0.038 (3)	0.037 (2)	0.041 (3)	0.002 (2)	−0.022 (2)	0.001 (2)
C5B	0.035 (2)	0.039 (2)	0.035 (2)	0.002 (2)	−0.016 (2)	−0.001 (2)
C6A	0.039 (3)	0.039 (2)	0.035 (3)	0.000 (2)	−0.012 (2)	−0.005 (2)
C6B	0.051 (3)	0.031 (2)	0.038 (3)	0.003 (2)	−0.015 (2)	0.002 (2)

### Geometric parameters (Å, °)

**Table:**

Br1—Zn	2.4275 (7)	N4B—C4B	1.323 (5)
Br2—Zn	2.4232 (7)	N1A—H1A	0.8600
Zn—N3A	2.060 (4)	N1B—H1B	0.8600
Zn—N3B	2.049 (3)	N4A—H3A	0.8600
O2A—C2A	1.233 (6)	N4A—H2A	0.8600
O2B—C2B	1.234 (5)	N4B—H2B	0.8600
N1A—C2A	1.365 (6)	N4B—H3B	0.8600
N1A—C6A	1.342 (6)	C4A—C5A	1.409 (7)
N1B—C2B	1.370 (5)	C4B—C5B	1.414 (6)
N1B—C6B	1.367 (6)	C5A—C6A	1.341 (6)
N3A—C2A	1.371 (5)	C5B—C6B	1.330 (6)
N3A—C4A	1.346 (6)	C5A—H5A	0.9300
N3B—C2B	1.371 (5)	C5B—H5B	0.9300
N3B—C4B	1.347 (5)	C6A—H6A	0.9300
N4A—C4A	1.324 (6)	C6B—H6B	0.9300
			
Br1···Br2	3.8306 (7)	N4A···Br2^ii^	3.339 (4)
Br1···O2B	3.558 (2)	N4A···C4A^iii^	3.325 (5)
Br1···N1B^i^	3.483 (3)	N4B···Br1^iv^	3.463 (3)
Br1···N4A	3.577 (4)	N4B···Br2	3.454 (3)
Br1···N4B^ii^	3.463 (3)	N4B···O2B^iv^	3.003 (5)
Br2···C5A^iii^	3.726 (4)	C2B···C6B^v^	3.588 (6)
Br2···N4A^iv^	3.339 (4)	C2B···N1B^v^	3.306 (5)
Br2···N4B	3.454 (3)	C2B···C2B^v^	3.592 (5)
Br2···C6B^i^	3.404 (5)	C4A···C6A^vii^	3.387 (6)
Br2···N3A	3.511 (3)	C4A···N4A^iii^	3.325 (5)
Br2···Br1	3.8306 (7)	C4A···C4A^iii^	3.520 (6)
Br2···O2A	3.488 (3)	C4B···O2A	3.116 (5)
Br1···H6B^v^	3.1100	C5A···N1A^vii^	3.357 (5)
Br1···H2A	2.7400	C5A···Br2^iii^	3.726 (4)
Br1···H2B^ii^	3.1900	C5A···Zn^iii^	4.148 (4)
Br1···H3B^ii^	3.0700	C5A···C6A^vii^	3.425 (6)
Br1···H1B^i^	2.7000	C5B···C5B^ix^	3.472 (6)
Br2···H2A^iv^	3.0900	C6A···C4A^vii^	3.387 (6)
Br2···H3A^iv^	2.9100	C6A···C5A^vii^	3.425 (6)
Br2···H6B^i^	3.2000	C6A···O2B^vii^	3.292 (6)
Br2···H2B	2.6500	C6B···O2B^v^	3.387 (6)
Br2···H3A^iii^	3.2200	C6B···C2B^v^	3.588 (6)
Br2···H5A^iii^	2.8700	C6B···Br2^viii^	3.404 (5)
Zn···C5A^iii^	4.148 (4)	C2A···H1A^vi^	2.8500
Zn···H2A	2.9100	C5B···H5B^ix^	3.0400
Zn···H2B	2.8800	H1A···O2A^vi^	1.9400
Zn···H5A^iii^	3.6300	H1A···C2A^vi^	2.8500
O2A···Br2	3.488 (3)	H1B···Br1^viii^	2.7000
O2A···N3B	2.915 (5)	H2A···Br1	2.7400
O2A···C4B	3.116 (5)	H2A···Br2^ii^	3.0900
O2A···N1A^vi^	2.766 (5)	H2A···Zn	2.9100
O2B···C6B^v^	3.387 (6)	H2B···Br1^iv^	3.1900
O2B···Br1	3.558 (2)	H2B···Br2	2.6500
O2B···N3A	3.257 (5)	H2B···Zn	2.8800
O2B···N4B^ii^	3.003 (5)	H3A···Br2^ii^	2.9100
O2B···C6A^vii^	3.292 (6)	H3A···H5A	2.4000
O2A···H1A^vi^	1.9400	H3A···Br2^iii^	3.2200
O2B···H5B^ii^	2.8600	H3B···Br1^iv^	3.0700
O2B···H3B^ii^	2.1900	H3B···O2B^iv^	2.1900
O2B···H6A^vii^	2.4200	H3B···H5B	2.3800
N1A···O2A^vi^	2.766 (5)	H5A···H3A	2.4000
N1A···C5A^vii^	3.357 (5)	H5A···Br2^iii^	2.8700
N1B···Br1^viii^	3.483 (3)	H5A···Zn^iii^	3.6300
N1B···C2B^v^	3.306 (5)	H5B···O2B^iv^	2.8600
N3A···Br2	3.511 (3)	H5B···H3B	2.3800
N3A···O2B	3.257 (5)	H5B···C5B^ix^	3.0400
N3A···N3B	3.295 (5)	H6A···O2B^vii^	2.4200
N3B···O2A	2.915 (5)	H6B···Br2^viii^	3.2000
N3B···N3A	3.295 (5)	H6B···Br1^v^	3.1100
N4A···Br1	3.577 (4)		
			
Br1—Zn—Br2	104.31 (2)	O2A—C2A—N1A	121.3 (4)
Br1—Zn—N3A	116.18 (9)	N1A—C2A—N3A	118.6 (4)
Br1—Zn—N3B	111.56 (10)	O2A—C2A—N3A	120.2 (4)
Br2—Zn—N3A	102.82 (9)	O2B—C2B—N3B	121.7 (3)
Br2—Zn—N3B	115.31 (9)	O2B—C2B—N1B	120.6 (4)
N3A—Zn—N3B	106.66 (13)	N1B—C2B—N3B	117.7 (4)
C2A—N1A—C6A	122.6 (4)	N3A—C4A—C5A	121.7 (4)
C2B—N1B—C6B	122.4 (4)	N3A—C4A—N4A	117.7 (4)
Zn—N3A—C2A	109.0 (3)	N4A—C4A—C5A	120.6 (4)
Zn—N3A—C4A	132.0 (3)	N4B—C4B—C5B	119.5 (4)
C2A—N3A—C4A	119.0 (4)	N3B—C4B—C5B	121.7 (4)
Zn—N3B—C2B	109.3 (2)	N3B—C4B—N4B	118.8 (4)
Zn—N3B—C4B	128.1 (3)	C4A—C5A—C6A	117.9 (4)
C2B—N3B—C4B	119.9 (3)	C4B—C5B—C6B	117.8 (4)
C2A—N1A—H1A	119.00	N1A—C6A—C5A	120.2 (4)
C6A—N1A—H1A	119.00	N1B—C6B—C5B	120.4 (4)
C2B—N1B—H1B	119.00	C4A—C5A—H5A	121.00
C6B—N1B—H1B	119.00	C6A—C5A—H5A	121.00
H2A—N4A—H3A	120.00	C4B—C5B—H5B	121.00
C4A—N4A—H2A	120.00	C6B—C5B—H5B	121.00
C4A—N4A—H3A	120.00	N1A—C6A—H6A	120.00
H2B—N4B—H3B	120.00	C5A—C6A—H6A	120.00
C4B—N4B—H2B	120.00	N1B—C6B—H6B	120.00
C4B—N4B—H3B	120.00	C5B—C6B—H6B	120.00
			
Br1—Zn—N3A—C2A	−176.9 (2)	C4A—N3A—C2A—O2A	−179.1 (4)
Br1—Zn—N3A—C4A	5.3 (4)	C4A—N3A—C2A—N1A	2.1 (6)
Br2—Zn—N3A—C2A	69.9 (3)	Zn—N3A—C4A—N4A	−5.4 (6)
Br2—Zn—N3A—C4A	−107.9 (4)	Zn—N3A—C4A—C5A	175.1 (3)
N3B—Zn—N3A—C2A	−51.9 (3)	C2A—N3A—C4A—N4A	177.1 (4)
N3B—Zn—N3A—C4A	130.4 (4)	C2A—N3A—C4A—C5A	−2.5 (6)
Br1—Zn—N3B—C2B	70.0 (3)	Zn—N3B—C2B—O2B	−13.9 (5)
Br1—Zn—N3B—C4B	−128.9 (3)	Zn—N3B—C2B—N1B	166.2 (3)
Br2—Zn—N3B—C2B	−171.3 (2)	C4B—N3B—C2B—O2B	−176.7 (4)
Br2—Zn—N3B—C4B	−10.2 (4)	C4B—N3B—C2B—N1B	3.3 (5)
N3A—Zn—N3B—C2B	−57.8 (3)	Zn—N3B—C4B—N4B	20.3 (5)
N3A—Zn—N3B—C4B	103.2 (3)	Zn—N3B—C4B—C5B	−160.4 (3)
C6A—N1A—C2A—O2A	−179.2 (4)	C2B—N3B—C4B—N4B	179.6 (4)
C6A—N1A—C2A—N3A	−0.4 (6)	C2B—N3B—C4B—C5B	−1.1 (6)
C2A—N1A—C6A—C5A	−1.0 (6)	N3A—C4A—C5A—C6A	1.2 (6)
C6B—N1B—C2B—O2B	175.8 (4)	N4A—C4A—C5A—C6A	−178.4 (4)
C6B—N1B—C2B—N3B	−4.2 (6)	N3B—C4B—C5B—C6B	−0.4 (6)
C2B—N1B—C6B—C5B	2.8 (7)	N4B—C4B—C5B—C6B	178.9 (4)
Zn—N3A—C2A—O2A	2.8 (5)	C4A—C5A—C6A—N1A	0.6 (6)
Zn—N3A—C2A—N1A	−176.0 (3)	C4B—C5B—C6B—N1B	−0.4 (7)

Symmetry codes: (i) *x*, *y*+1, *z*; (ii) *x*−1, *y*, *z*; (iii) −*x*+1, −*y*+2, −*z*+1; (iv) *x*+1, *y*, *z*; (v) −*x*+1, −*y*+1, −*z*+2; (vi) −*x*+2, −*y*+1, −*z*+1; (vii) −*x*+1, −*y*+1, −*z*+1; (viii) *x*, *y*−1, *z*; (ix) −*x*+2, −*y*+1, −*z*+2.

### Hydrogen-bond geometry (Å, °)

**Table:**

*D*—H···*A*	*D*—H	H···*A*	*D*···*A*	*D*—H···*A*
N1A—H1A···O2A^vi^	0.86	1.94	2.766 (5)	161
N1B—H1B···Br1^viii^	0.86	2.70	3.483 (3)	151
N4A—H2A···Br1	0.86	2.74	3.577 (4)	165
N4B—H2B···Br2	0.86	2.65	3.454 (3)	155
N4A—H3A···Br2^ii^	0.86	2.91	3.339 (4)	112
N4B—H3B···O2B^iv^	0.86	2.19	3.003 (5)	157
C5A—H5A···Br2^iii^	0.93	2.87	3.726 (4)	153
C6A—H6A···O2B^vii^	0.93	2.42	3.292 (6)	156

Symmetry codes: (vi) −*x*+2, −*y*+1, −*z*+1; (viii) *x*, *y*−1, *z*; (ii) *x*−1, *y*, *z*; (iv) *x*+1, *y*, *z*; (iii) −*x*+1, −*y*+2, −*z*+1; (vii) −*x*+1, −*y*+1, −*z*+1.
